# Causal effect of COVID-19 on optic nerve and visual pathway disorders: genetic evidence of lung-brain axis

**DOI:** 10.3389/fimmu.2024.1440262

**Published:** 2024-07-16

**Authors:** Chunge Cao, Qiong Li, Dajun Cai, Chaoyan Yue, Hu Zhao

**Affiliations:** ^1^ Department of Obstetrics and Gynecology, Second Affiliated Hospital of Zhengzhou University, Zhengzhou, China; ^2^ Department of Obstetrics and Gynecology, First People’s Hospital of Chenzhou, Chenzhou, China; ^3^ Department of Laboratory Medicine, Obstetrics and Gynecology Hospital of Fudan University, Shanghai, China

**Keywords:** COVID-19, optic nerve, visual pathways, Mendelian randomization, lung-brain axis

## Abstract

**Purpose:**

To investigate the potential causal association between COVID-19 exposure and optic nerve and visual pathway disorders through a two-sample bidirectional Mendelian randomization (MR) analysis, and to provide empirical support for the lung-brain axis.

**Methods:**

This MR analysis utilized publicly accessible summary-level data from genome-wide association studies on COVID-19 (n=158,783) and optic nerve and visual pathway diseases (n=412,181), primarily involving individuals of European descent. The random-effect inverse-variance weighted estimation was applied as the main analytical approach, complemented by MR-Egger, weighted median, and weighted mode methods. The heterogeneity and pleiotropy of the instrumental variables were assessed using Cochran’s Q test, leave-one-out sensitivity analysis, MR-Egger intercept test, MR-PRESSO, and funnel plot evaluations.

**Results:**

In the forward analysis, the inverse-variance weighted method identified a significant causal effect of COVID-19 on optic nerve and visual pathway disorders (odds ratio = 1.697, 95% confidence interval: 1.086–2.652, *p* = 0.020). Directionally consistent results were also observed with MR-Egger regression, weighted median, and weighted mode approaches. Conversely, the reverse analysis revealed no causal effects of optic nerve and visual pathway disorders on COVID-19 susceptibility.

**Conclusion:**

Our findings suggest that COVID-19 exposure may increase the risk of developing optic nerve and visual pathway disorders, supporting the lung-brain axis hypothesis. These results underscore the importance of vigilant monitoring of the visual system in patients recovering from COVID-19 and suggest potential avenues for future therapeutic strategies.

## Introduction

The COVID-19 pandemic has triggered a worldwide health emergency with far-reaching repercussions, surpassing the initial respiratory symptoms to include prolonged neurological complications, often termed ‘long COVID’ (1). Observational studies have potentially shown that COVID-19 can lead to disorders of optic nerve and visual pathways, including uveitis, optic neuritis, macular degeneration, and retinal vascular diseases (2, 3). These conditions may manifest as a variety of symptoms, such as vision loss, visual field defects, reduced sensitivity to light and contrast, as well as alterations in color perception (4).

The burgeoning concept of the lung-brain axis delineates a sophisticated biological network facilitating communication between the lungs and brain via neural, inflammatory, immune, and neuroendocrine signaling pathways (5). The central nervous system (CNS) plays a critical role in modulating the pulmonary response to stress and inflammation through neuroendocrine mechanisms (6). Brain injuries may provoke pulmonary complications by eliciting the release of necrotic substances (7), while pulmonary conditions have the potential to instigate cerebrovascular diseases through the induction of white matter lesions (8). Additionally, the significant influence of the lung microbiome on brain autoimmunity has been increasingly acknowledged (9). This reciprocal communication highlights the symbiotic interplay between respiratory and neurological health. Early hypotheses suggested that the SARS-CoV-2 might penetrate the CNS via the nasal cavity and olfactory pathway or the blood-brain barrier (BBB) (10, 11). However, cerebrospinal fluid analyses in patients presenting neuropsychiatric symptoms have revealed minimal detection of viral RNA, with only 8.6% (3 out of 35) identified through reverse transcription polymerase chain reaction (12). This suggests that neurological symptoms do not typically result from direct SARS-CoV-2 infection of the brain tissue.

Although there has been considerable research, the connection between COVID-19 and optic nerve or visual pathway disorders is yet to be conclusively proven, hindered by insufficient large-scale cohort studies and conclusive evidence. Concurrently, the varied symptoms experienced by individuals with Long COVID, coupled with the challenge of distinguishing symptoms caused by COVID-19 from those that are aggravations of existing or sporadic illnesses, significantly complicate the understanding of underlying mechanisms and the development of therapeutic strategies.

Mendelian randomization (MR) is an innovative approach in epidemiology, utilizing genetic variants as proxies for deducing the causal impact of various exposures on outcomes (13). These genetic markers are randomly segregated and allocated during the formation of gametes and at conception, remaining uninfluenced by the development or progression of the outcome (14). As a result, MR typically protects against biases and unmeasured confounding factors, providing a more robust basis for causal deduction than is possible with traditional observational studies (15). In this study, we aim to investigate the causal link between COVID-19 and disorders of optic nerve and visual pathways through the MR method, to assess its impact size, and to furnish evidence supporting the lung-brain axis hypothesis.

## Materials and methods

### Study design

**Figure 1:**
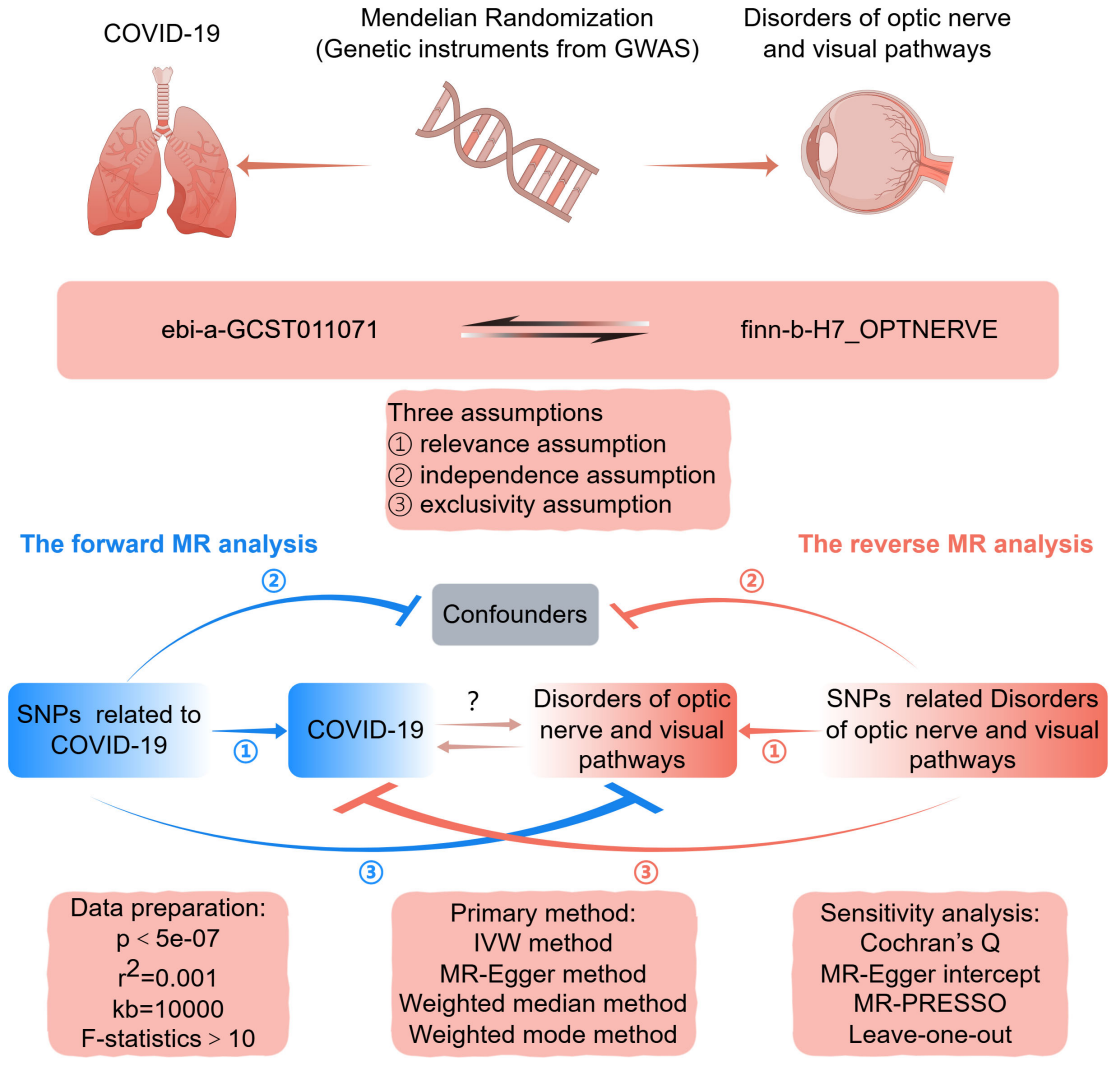
Graphical abstract for this MR study between COVID-19 and disorders of optic nerve and visual pathways. GWAS, genome-wide association study; MR, Mendelian randomization; SNP, single nucleotide polymorphism; IVW, inverse-variance weighted. By FigDraw.

**Figure 1** depicts a graphical abstract illustrating the bidirectional MR study. The forward MR assessed the causal effect of COVID-19 on disorders of optic nerve and visual pathways. The reverse MR assessed the causal effect of optic nerve and visual pathway disorders on COVID-19 susceptibility. This study used datasets from extensive genome-wide association studies (GWAS). Single-nucleotide polymorphisms (SNPs) from these GWAS datasets served as instrumental variables (IVs) for the exposure. The MR analysis is based on three critical assumptions: firstly, the IVs are strongly associated with the exposure; secondly, the IVs are related to the outcome solely through the exposure under investigation; and thirdly, the IVs are independent of any confounding factors (16). This research employed publicly accessible, summary-level GWAS data from studies that had previously received institutional review board approval. Additional ethical approval or informed consent was not requisite for this study’s data usage, given its public, anonymized, and de-identified nature.

### Data sources

The GWAS datasets for COVID-19 (GWAS ID: ebi-a-GCST011071) and optic nerve and visual pathway disorders (GWAS ID: finn-b-H7_OPTNERVE) came from the IEU Open GWAS Project. The COVID-19 dataset included 29,071 cases and 1,559,712 controls, with a total of 8,103,014 SNPs. The dataset for optic nerve and visual pathway disorders comprised 1,301 cases and 217,491 controls, encompassing 16,380,466 SNPs. The majority of participants in both GWAS datasets were of European descent.

### Selection of IVs

We first selected SNPs that were strongly associated with the exposure, using a genome-wide significance threshold of *p* < 5e-08. To ensure a sufficient number of SNPs for the exposure, we allowed for a relaxed threshold of *p* < 5e-07 or *p* < 5e-06. We used the European ancestry data from the 1000 Genomes Project (RRID: SCR_008801) and employed stringent clumping parameters with a distance greater than 10,000 kb and an (r^2^ < 0.001) to minimize linkage disequilibrium among the variables. To enhance the accuracy of our results, we excluded palindromic SNPs with intermediate allele frequencies. Potential confounders were identified and removed by querying the PhenoScanner V2 database for SNPs associated with possible confounding factors. Weak IVs were discarded using the F statistic to ensure a robust association between the exposure factors and the IVs. The F statistic was determined by the squared beta coefficient divided by its variance for the SNP-exposure association, with an F statistic greater than 10 indicating a strong association (17).

### MR analysis

Assessing the causal link between COVID-19 and disorders of optic nerve and visual pathways, we employed four MR methods: the random-effect inverse-variance weighted (IVW) estimation method, MR-Egger regression method, weighted-median estimator method, and weighted mode-based method. The IVW method, when directional pleiotropy is absent, offers a more stable and precise estimation of causal effects by integrating the Wald ratio estimates from each instrumental variant (18, 19). Consequently, IVW served as the primary method for this study, while the other three methods served as supplementary analyses. Consistent results across all four methods bolster the credibility of the causality estimates. In cases of discrepancy, the IVW outcome is given precedence as the principal finding. However, we consider the IVW results reliable only if they are directionally concordant with the findings of the supplementary methods.

### Sensitivity analysis

The Cochran’s Q test was applied to assess the heterogeneity among IVs, with a *p*-value below 0.05 indicating significant heterogeneity. A leave-one-out sensitivity analysis was performed to ensure no single SNP disproportionately influenced the causal estimate. This involved sequentially discarding each SNP associated with the exposure and repeating the IVW analysis to verify the stability of the causal effects of each SNP. The MR-Egger intercept test was employed to detect and adjust for bias from directional pleiotropy; a non-zero intercept suggests the presence of such bias (20). Additionally, the MR-PRESSO method was employed to identify and correct for horizontal pleiotropy; a global test resulting in a *p*-value under 0.05 signifies horizontal pleiotropy between IVs and outcomes; the outlier test pinpoints SNPs that may be outliers, potentially violating MR assumptions, necessitating their exclusion from the analysis (21).

### Statistical analysis

The MR analysis used TwoSampleMR (version 4.3.1), an R statistical software package that facilitates the two-sample MR approach. Causal estimates were presented as odds ratios (ORs) with 95% confidence intervals (CIs). Statistical significance was determined by a two-tailed p-value of less than 0.05.

## Results

### Genetic instruments and strength

In our bidirectional analysis, we identified nine SNPs significantly associated with COVID-19, showing a genome-wide significance threshold of *p*<5e-07, and fourteen SNPs linked to disorders of optic nerve and visual pathways, with a threshold of *p*<5e-06. These associations were established after linkage disequilibrium clumping, data harmonization, and mining of the Phenoscanner V2 database, as detailed in **Supplementary Tables 1**, **2**. The F-statistics for each SNP were above 10, indicating a negligible risk of weak instrument bias.

### The causal effect of COVID-19 on optic nerve and visual pathway disorders

**Figure 2:**
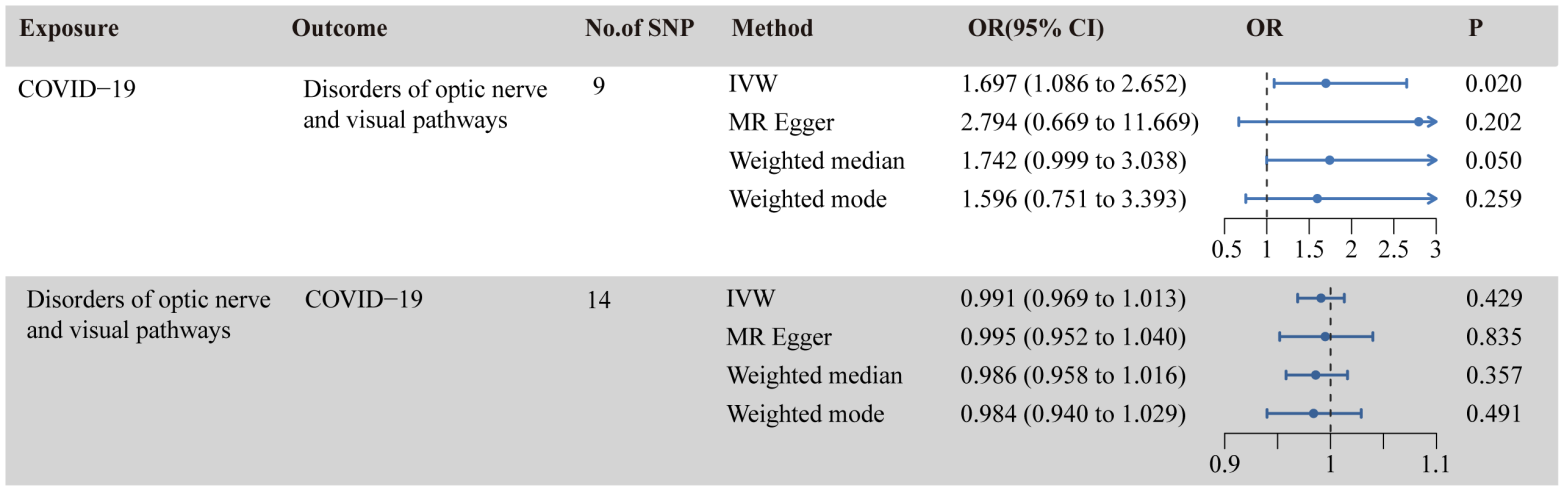
Causal relationship between COVID-19 and disorders of optic nerve and visual pathways in the MR analyses. SNPs, single-nucleotide polymorphisms; OR, odds ratio; CI, confidence interval; IVW, inverse-variance weighted; MR, Mendelian randomization.

**Figure 3:**
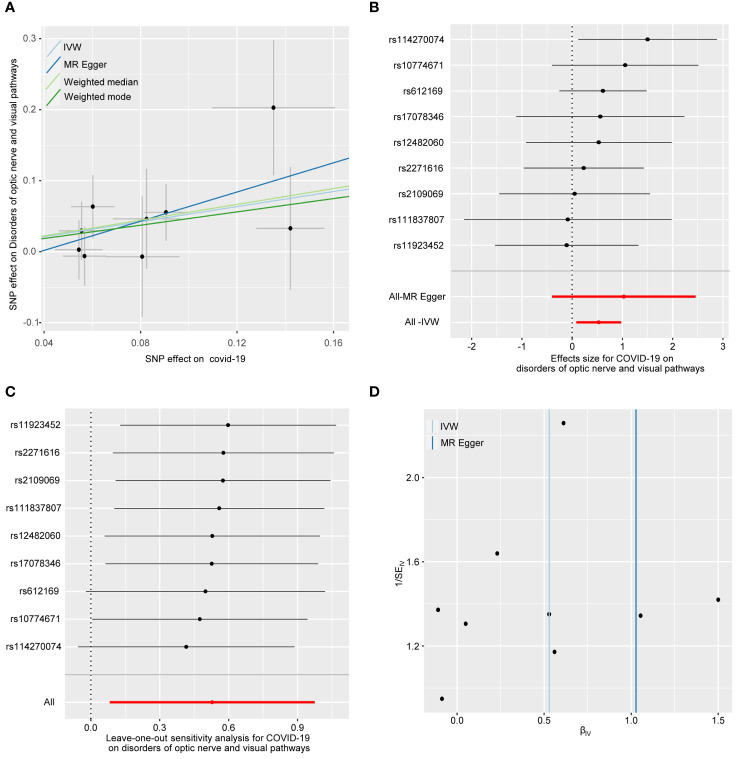
The forward MR analyses: Casual effect of COVID-19 on disorders of optic nerve and visual pathways. **(A)** Scatter plot of the association between COVID-19 and disorders of optic nerve and visual pathways. **(B)** Forest plot to show the causal effect size estimate of COVID-19 on disorders of optic nerve and visual pathways (red line segment) and 95% CI values (gray line segment) for each SNP. **(C)** Leave-one-out analyses to evaluate the impact of each SNP on the overall result. **(D)** Funnel plot to detect obvious heterogeneity and system bias. IVW, inverse variance weighted; SNPs, single-nucleotide polymorphisms.

**Figure 2** illustrates the causal relationship between COVID-19 and the incidence of optic nerve and visual pathway disorders. The IVW method indicates a notable causal influence of COVID-19 on optic nerve and visual pathway disorders, with an OR of 1.697 and a 95% CI ranging 1.086 to 2.652 (*p* = 0.020). Complementary methods, including MR-Egger, Weighted Median, and Weighted Mode, yielded directionally consistent but not statistically significant results compared to IVW. The robustness of these findings is supported by sensitivity analyses presented in **Table 1** and **Figure 3**. Cochran’s Q test indicates uniformity across SNP effect estimates (*p* = 0.841), and the MR-Egger intercept negates the presence of directional pleiotropy (intercept = -0.039, *p* = 0.495). The MR-PRESSO test also finds no evidence of horizontal pleiotropy or outliers (*p* = 0.866), and the leave-one-out analysis substantiates the consistency of our results.

**Table 1:** Results of pleiotropy and heterogeneity analyses.

Exposure	Outcome	Cochran’s Q test		MR-Egger test		MR-PRESSO test	
		IVW Q	*p*-value	Intercept	*p*-value	Global test *p*	Outliers
COVID-19	Disorders of optic nerve and visual pathways	4.177	0.841	-0.039	0.495	0.866	None
Disorders of optic nerve and visual pathways	COVID-19	5.336	0.967	-0.001	0.835	0.967	None

IVW, inverse-variance weighted; SNPs, single‐nucleotide polymorphisms.

### The causal effect of optic nerve and visual pathway disorders on COVID-19

**Figure 4:**
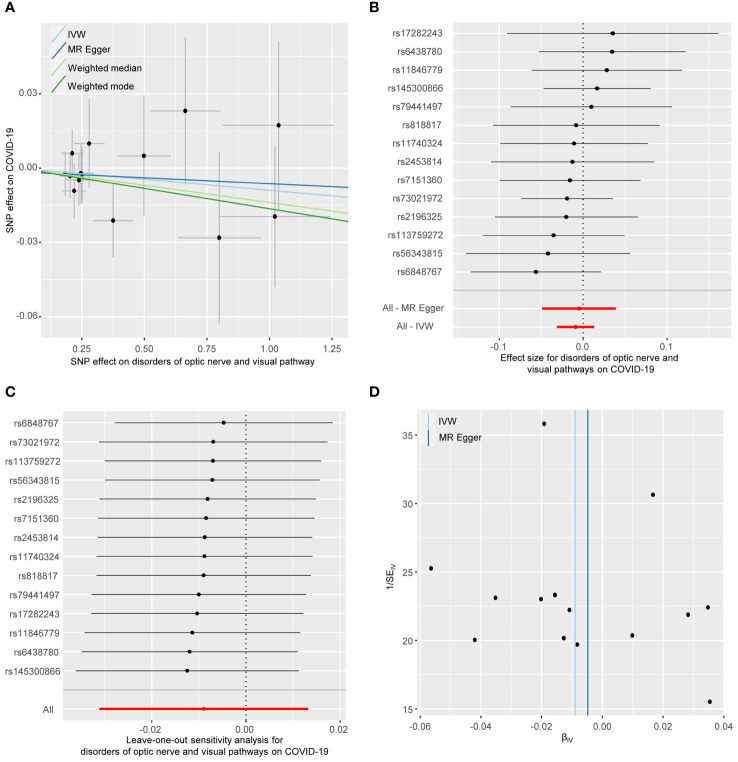
The reverse MR analyses: Casual effect of disorders of optic nerve and visual pathways on COVID-19. **(A)** Scatter plot of the association between disorders of optic nerve and visual pathways and COVID-19. **(B)** Forest plot to show the causal effect size estimate of disorders of optic nerve and visual pathways on COVID-19 (red line segment) and 95% CI values (gray line segment) for each SNP. **(C)** Leave-one-out analyses to evaluate the impact of each SNP on the overall result. **(D)** Funnel plot to detect obvious heterogeneity and system bias. IVW, inverse variance weighted; SNPs, single-nucleotide polymorphisms.

As shown in **Figure 2**, the IVW analysis suggests that optic nerve and visual pathway disorders do not causally affect COVID-19 susceptibility (OR = 0.991, 95% CI: 0.969-1.012; *p* = 0.429). This finding is supported by MR-Egger, Weighted Median, and Weighted Mode methods. Detailed in **Table 1** and **Figure 4**, the sensitivity analyses affirm the reliability of our conclusions. Cochran’s Q test confirms no variability among the SNP effect estimates (*p* = 0.967), and the MR-Egger intercept indicates an absence of directional pleiotropy (Intercept = -0.001, *p* = 0.835). Additionally, the MR-PRESSO test reveals no horizontal pleiotropy or outliers (*p* = 0.967), and the leave-one-out test reinforces the dependability of our findings.

## Discussion

In this study, we employed a two-sample bidirectional MR approach to explore the relationship between COVID-19 and disorders of optic nerve and visual pathways. The forward results provide compelling genetic evidence that COVID-19 may increase the risk of disorders of optic nerve and visual pathways. The reverse results show that disorders of optic nerve and visual pathways have no causal effect on COVID-19 susceptibility. Sensitivity analyses have reinforced the robustness of our results.

Our research has unearthed critical insights that could be transformative for clinical practice and public health policy. These findings highlight a significant correlation between viral infections and an increased risk of optic nerve and visual pathway disorders. Such knowledge is vital for physicians, enabling them to diagnose and treat these conditions more proactively, which may lead to better patient outcomes. Moreover, our study supports the need for public health authorities to intensify visual health monitoring and preventive actions during the pandemic, with a particular focus on vulnerable groups. Educating the public and patients about the ocular risks linked to COVID-19 is essential, as it encourages early medical intervention when symptoms are detected. This research also marks a pivotal step in understanding the lung-brain axis, potentially reshaping our comprehension of how respiratory infections influence neurological health. It paves the way for further investigations into the specific mechanisms by which COVID-19 impacts the optic nerve and visual pathways, spurring the creation of novel prophylactic and therapeutic approaches. In addition, these discoveries could be instrumental in guiding the development of vaccines and shaping public health strategies aimed at mitigating the long-term sequelae of COVID-19.

A comprehensive retrospective case-control study involving 2,351 patients revealed a notable increase in immune-mediated ocular conditions like uveitis and optic neuritis, potentially linked to COVID-19 (2). Concurrently, an observational cross-sectional study indicated that patients with neurological symptoms of COVID-19 exhibited a decrease in the thickness of the retinal nerve fiber layer and ganglion cell complex, as well as a reduced vessel density (22). In contrast, other research indicated an elevation in the thickness of the retinal nerve fiber layer, implying potential inflammation of the optic nerve or temporary alterations during acute COVID-19 infection (23). Additionally, a separate study observed increased intraocular pressure and specific changes in the outer retina in severe cases 80 days post-COVID-19 infection, although no evidence of uveitis was detected (24). The discrepancies observed across these studies could be attributed to a variety of factors, including study design, methodological variances, sample size differences, reporting biases, the timing of assessments, the expertise of the investigators, the specific definitions of disorders, and the level of control over confounding variables.

Several host receptors facilitate the entry of SARS-CoV-2 into human cells, with the angiotensin-converting enzyme 2 (ACE2) receptor being paramount. The viral spike protein (S protein) binds to ACE2, triggering the cleavage of the S protein into S1 and S2 subunits. The S1 subunit, containing the receptor binding domain, attaches to ACE2, and subsequent cleavage of the S2 subunit by host proteases facilitates membrane fusion and viral entry (25). Multiple tissues including mucosa of the nose, mouth, and eyes, respiratory tract, lungs, heart, liver, kidney, brain, gastrointestinal tract, placenta, and other organs express ACE2 with a high level (10). Current researches indicate that SARS-CoV-2 could invade the human brain through multiple pathways (26). Initially, the virus may progress from the nasal cavity to the olfactory bulb via olfactory nerves. Secondly, the virus might access the bloodstream via damaged respiratory epithelium and proceed to penetrate the BBB, utilizing ACE2-mediated transcellular pathways or disrupting the barrier’s tight-junctions. Lastly, the virus has the potential to invade ocular tissues and navigate along the optic nerve to the occipital cortex. However, CSF testing in patients with COVID-19 to find evidence of viral neuroinvasion by SARS-CoV-2 showed that of 304 patients whose CSF was tested for SARS-CoV-2 viral RNA, there were 17 (6%) whose test was positive, all of whom had symptoms that localized to the CNS, of 58 patients whose CSF was tested for SARS-CoV-2 antibody, 7 (12%) had positive antibodies with evidence of intrathecal synthesis, all of whom had symptoms that localized to the CNS, of 132 patients who had oligoclonal bands evaluated, 3 (2%) had evidence of intrathecal antibody synthesis (27). The above results indicate that most neurological complications associated with SARS- CoV-2 are unlikely to be related to direct viral neuroinvasion.

Recent studies have illuminated the lung-brain axis, which allows two-way communication between the lungs and CNS. This axis consists of the following multiple interrelated pathways. As part of the autonomic nervous system, neuroanatomical pathway involves neurons and cells for respiratory communication, involving the phrenic nerve for breathing and the vagus nerve for involuntary functions. Pulmonary receptors coordinate with the brain to regulate breathing, with increased sensitivity in diseases like asthma and COPD causing dyspnea. Quick signaling of this pathway is essential for cough reflexes and adjusting respiration to maintain homeostasis and adapt to health changes. In the endocrine pathway, the hypothalamus-pituitary-adrenal axis releases glucocorticoids during lung stress or inflammation, while the sympathetic system secretes adrenaline and noradrenaline for ‘fight or flight’ responses. They work together to maintain balance and respond to diseases. In immune pathway, CNS conditions can cause lung damage via cytokines, and lung infections can negatively affect the CNS. Systemic inflammation is linked to CNS issues like paraneoplastic syndromes and autoimmune diseases. The lung serves as a critical site for the reactivation of autoreactive T cells, which can then migrate to the CNS and trigger autoimmune disorders. The lung’s microbial balance affects CNS susceptibility to autoimmune conditions. Metabolites and microorganisms pathway involves the transfer of biological substances like exosomes and outer-membrane vesicles, ferrying proteins, lipids, nucleic acids, and other bioactive molecules between the CNS and lungs, crossing the BBB. Exosomes can carry cytokines that intensify lung injury and affect microglial activity in the brain, impacting conditions like Alzheimer’s disease. Similarly, outer-membrane vesicles from bacteria can provoke central neuropathy and neuroinflammatory diseases. The gas pathway is crucial for how respiratory gases impact the CNS. Air pollutants, like ozone, can indirectly influence CNS functions by altering neuronal activity and activating stress response pathways, which may result in cognitive and behavioral changes. Diseases of the lungs that lead to hypoxemia and hypercapnia are associated with CNS disorders. Chronic hypoxemia is known to cause white matter changes that are associated with Alzheimer’s disease. On the other hand, mild hypercapnia might offer neuroprotection, while severe hypercapnia can aggravate brain injury (28).

In a prospective study assessing the prevalence of serum myelin oligodendrocyte glycoprotein antibody (MOG-Ab) and aquaporin-4 antibody (AQP4-Ab) among 35 patients with clinical optic neuritis and confirmed COVID-19, it was found that serum MOG-Ab and AQP4-Ab were detected in 28.6% (10/35) and 5.7% (2/35) optic neuritis cases after COVID-19 (29). Positivity for MOG-Ab is generally indicative of MOG antibody-associated disease, an immune-mediated condition that demyelinates the optic nerves, brain, and spinal cord (30). AQP4-Ab positivity is a strong marker for neuromyelitis optica spectrum disorder, a severe autoimmune inflammatory disorder of the CNS, marked by intense optic neuritis and myelitis (31). The identification of these antibodies, coupled with a favorable response to pulse steroid therapy, suggests that optic neuritis related to COVID-19 represents a post-infectious, immune-mediated inflammatory response (29). Furthermore, a comprehensive multicenter study has shown that recipients of the mRNA vaccines BNT162b2 and mRNA-1273 have a notably higher risk of retinal vascular occlusion within two years post-vaccination, suggesting that SARS-CoV-2 may cause neuro-ophthalmic damage via mechanisms beyond direct viral invasion (32).

Integrating the available evidence, we propose that SARS-CoV-2 may precipitate conditions affecting the optic nerve and visual pathways via the lung-brain axis routes. Initially, SARS-CoV-2 might penetrate the CNS through BBB, directly targeting neurons within the optic nerve and visual pathways. Furthermore, lung inflammation from SARS-CoV-2 could amplify the release of inflammatory mediators like cytokines, enhancing BBB permeability and leading to optic neuritis and retinal vascular occlusion. Additionally, lung impairment due to SARS-CoV-2 might disrupt oxygen and carbon dioxide exchange, compromising the oxygenation of the brain and optic nerve, with sustained hypoxemia and hypercapnia potentially causing damage to these structures. Moreover, the infection could disrupt lung microbial homeostasis and metabolic outputs, which might, via the bloodstream, impact the optic nerve and visual pathways’ functionality. SARS-CoV-2 could also present antigens mimicking MOG or AQP4 proteins on astrocytes, eliciting a pathogenic T cell response and antigen-antibody reaction, leading to inflammation and demyelination, thereby impairing vision. A less likely but possible pathway for the generation of MOG-Ab may involve the incidental unveiling of MOG protein to the immune system’s antigen-presenting cells during inflammation in the CNS’s white matter or optic nerve, instigated by COVID-19. The specific mechanisms by which COVID-19 causes disorders of optic nerve and visual pathways require further and more in-depth research.

Our study presents several notable strengths. Primarily, the MR approach we employed was less prone to confounding factors, such as inflammation, vascular disease, and tumor compression, which could also lead to optic nerve and visual pathway disorders. This robustness stems from our utilization of multiple COVID-19-associated SNPs, derived from extensive GWAS, as IVs. We further refine our analysis by excluding SNPS associated with potential confounders, identified through the PhenoScanner V2 database, to negate the impact of these confounders on our results. These measures provided substantial statistical power to establish causal relationships. Moreover, we implemented stringent criteria for the selection of IVs to uphold the foundational assumptions of MR, thereby mitigating the risk of weak instrument bias. To account for any anomalies induced by horizontal or directional pleiotropy, we utilized advanced methods such as MR-Egger and MR-PRESSO for detection and correction. Additionally, we confined the genetic background of our participants predominantly to European ancestry, which curtailed potential confounding effects arising from a more diverse population mix.

Our study has several limitations that warrant consideration. The datasets analyzed were primarily composed of individuals of European descent, potentially limiting the generalizability of our findings across different ethnic groups. Furthermore, the ongoing mutation of SARS-CoV-2 could influence factors such as viral transmissibility, viral load, disease severity, and the virus’s ability to evade immune responses. These mutations may complicate the causal inference regarding disorders of optic nerve and visual pathways. Currently, there is a lack of GWAS data on post-mutation SARS-CoV-2. Should such data become available for various SARS-CoV-2 strains in the future, we aim to conduct further investigations. Additionally, our MR analysis is based on publicly accessible summary-level data rather than individual-level data, precluding us from performing subgroup analyses based on COVID-19 severity. This limitation may result in less precise causal estimates and could impact the interpretation and generalization of our results.

## Conclusions

In summary, this MR study provides evidence suggesting that COVID-19 may elevate the risk of developing optic nerve and visual pathway disorders. Beyond deepening our comprehension of the interplay between COVID-19 and diseases of optic nerve and visual pathways, this research also introduces fresh perspectives and robust data to bolster lung-brain axis studies. These insights are pivotal for devising preventive measures and therapeutic interventions for nervous system diseases associated with infections. Moreover, they are instrumental in enhancing the quality of clinical care delivered to patients. Additionally, these discoveries lay the groundwork for the development of innovative therapeutic approaches for patients with infections.

## Data availability statement

The original contributions presented in the study are included in the article/**Supplementary Material**. Further inquiries can be directed to the corresponding authors.

## Ethics statement

Ethics approval and consent to participate were waived for this study because this study used publicly available summary-level GWAS data from published studies that had obtained institutional review board approval for their respective studies, and the data was public, anonymized, and de-identified.

## Author contributions

CC: Conceptualization, Funding acquisition, Investigation, Methodology, Project administration, Writing – original draft. QL: Data curation, Formal analysis, Writing – review & editing. DC: Conceptualization, Methodology, Writing – review & editing. CY: Data curation, Formal analysis, Methodology, Software, Supervision, Visualization, Writing – review & editing. HZ: Funding acquisition, Resources, Validation, Writing – review & editing.
