# Supplementary material for: Causal effect of COVID-19 on optic nerve and visual pathway disorders: genetic evidence of lung-brain axis
Source: Front Immunol. 2024 Jul 16;15:1440262. doi: 10.3389/fimmu.2024.1440262 (PMC11286426; doi:10.3389/fimmu.2024.1440262)
Supplement: Supplementary file 1 [file Table_1.docx]

**Supplementary materials**

**Table S1.** List of SNPs used as instrumental variables for COVID-19.

| SNP | Chr:position | EA | OA | EAF | F-Statistic | Association with exposure | | Association with outcome | |
| --- | --- | --- | --- | --- | --- | --- | --- | --- | --- |
|  |  |  |  |  |  | Beta(SE) | *p*-value | Beta(SE) | *p*-value |
| rs11923452 | 3:101433515 | C | T | 0.352 | 39.297 | -0.057(0.009) | 3.64E-10 | 0.006(0.041) | 0.881 |
| rs17078346 | 3:45845748 | C | A | 0.106 | 34.569 | 0.083(0.014) | 4.11E-09 | 0.046(0.070) | 0.511 |
| rs2271616 | 3:45838013 | T | G | 0.117 | 100.907 | 0.142(0.014) | 9.63E-24 | 0.033(0.087) | 0.705 |
| rs111837807 | 6:31121232 | C | T | 0.092 | 27.472 | 0.081(0.015) | 1.60E-07 | -0.007(0.085) | 0.934 |
| rs612169 | 9:136143442 | G | A | 0.353 | 99.976 | 0.091(0.009) | 1.54E-23 | 0.055(0.040) | 0.167 |
| rs10774671 | 12:113357193 | A | G | 0.672 | 44.884 | 0.06(0.009) | 2.09E-11 | 0.064(0.045) | 0.156 |
| rs114270074 | 16:56135555 | A | G | 0.035 | 28.128 | 0.135(0.025) | 1.14E-07 | 0.203(0.095) | 0.033 |
| rs2109069 | 19:4719443 | A | G | 0.316 | 30.869 | 0.054(0.01) | 2.76E-08 | 0.003(0.042) | 0.948 |
| rs12482060 | 21:34611571 | G | C | 0.342 | 34.799 | 0.056(0.009) | 3.66E-09 | 0.029(0.041) | 0.475 |

Abbreviations: SNP: single nucleotide polymorphism; Chr: chromosome; EA: effect allele; OA: other allele; EAF: effect allele frequency; SE: standard error.

**Table S2.** List of SNPs used as instrumental variables for disorders of optic nerve and visual pathways.

| SNP | Chr:position | EA | OA | EAF | F-Statistic | Association with exposure | | Association with outcome | |
| --- | --- | --- | --- | --- | --- | --- | --- | --- | --- |
|  |  |  |  |  |  | Beta(SE) | *p*-value | Beta(SE) | *p*-value |
| rs145300866 | 1:207638117 | C | T | 0.010 | 21.760 | 1.037(0.222) | 3.08E-06 | 0.017(0.034) | 0.609 |
| rs79441497 | 2:140730955 | G | A | 0.039 | 21.957 | 0.498(0.106) | 2.80E-06 | 0.005(0.024) | 0.841 |
| rs56343815 | 3:54239622 | C | A | 0.251 | 22.573 | -0.22(0.046) | 2.04E-06 | 0.009(0.011) | 0.401 |
| rs6438780 | 3:122901055 | C | A | 0.023 | 22.348 | -0.664(0.141) | 2.28E-06 | -0.023(0.030) | 0.436 |
| rs818817 | 3:38350543 | G | A | 0.822 | 22.015 | 0.247(0.053) | 2.72E-06 | -0.002(0.013) | 0.871 |
| rs2196325 | 4:182460379 | C | G | 0.185 | 21.471 | 0.239(0.052) | 3.60E-06 | -0.005(0.010) | 0.643 |
| rs6848767 | 4:469347 | G | A | 0.070 | 22.063 | 0.375(0.08) | 2.64E-06 | -0.021(0.015) | 0.154 |
| rs11740324 | 5:53491838 | C | T | 0.161 | 21.224 | 0.252(0.055) | 4.03E-06 | -0.003(0.011) | 0.810 |
| rs2453814 | 5:2493280 | C | A | 0.454 | 21.114 | -0.184(0.04) | 4.43E-06 | 0.002(0.009) | 0.798 |
| rs113759272 | 9:131300832 | C | T | 0.017 | 22.750 | 0.799(0.168) | 1.85E-06 | -0.028(0.035) | 0.416 |
| rs17282243 | 13:21839589 | T | C | 0.125 | 21.079 | -0.28(0.061) | 4.40E-06 | -0.010(0.018) | 0.582 |
| rs11846779 | 14:81615734 | C | T | 0.684 | 24.195 | 0.212(0.043) | 8.65E-07 | 0.006(0.010) | 0.536 |
| rs7151360 | 14:68902707 | G | T | 0.379 | 24.516 | 0.204(0.041) | 7.34E-07 | -0.003(0.009) | 0.716 |
| rs73021972 | 19:23749873 | C | T | 0.009 | 21.168 | 1.021(0.222) | 4.21E-06 | -0.020(0.029) | 0.493 |
